# Soluble and Insoluble Dietary Fibre in Date Fruit Varieties: An Evaluation of Methods and Their Implications for Human Health

**DOI:** 10.3390/foods12061231

**Published:** 2023-03-13

**Authors:** Lily Stojanovska, Habiba I. Ali, Afaf Kamal-Eldin, Usama Souka, Ayesha S. Al Dhaheri, Leila Cheikh Ismail, Serene Hilary

**Affiliations:** 1 Department of Nutrition and Health, College of Medicine and Health Sciences, United Arab Emirates University, Al Ain P.O. Box 15551, United Arab Emirates; 2 Institute for Health and Sport, Victoria University, Melbourne, VIC 3011, Australia; 3 Department of Food Science, College of Agriculture and Veterinary Medicine, United Arab Emirates University, Al Ain P.O. Box 15551, United Arab Emirates; 4 Department of Clinical Nutrition and Dietetics, College of Health Sciences, University of Sharjah, Sharjah P.O. Box 27272, United Arab Emirates; 5 Nuffield Department of Women’s and Reproductive Health, University of Oxford, Oxford OX1 2JD, UK

**Keywords:** dates, AOAC 991.43, dietary fibre, IDF, SDF, TDF, ANKOM, enzymatic gravimetric method

## Abstract

Dietary fibre analysis is expensive due to its reliance on enzymes such as α-amylase, protease, and amyloglucosidase. This study investigated whether enzymes are essential in analysing insoluble, soluble, and total dietary fibre (IDF, SDF and TDF) contents in dry fruits with very low starch and protein contents. The IDF, SDF, and TDF were measured in date fruits using the enzymatic gravimetric method AOAC 991.43 in the ANKOM dietary fibre analyser, with and without enzymatic digestion. The study analysed six date fruit varieties with a range of texture profiles. Our results highlighted agreement between both methods in the measured IDF, SDF, and TDF values. TDF values in date fruit varieties varied considerably, from 5.67% g/100 g to 10.33% g/100 g. Results from both methods also indicate that IDF constituted the bulk of dietary fibre content in all date fruit varieties (77.8% to 91.6%), while the proportion of SDF was between 8.4% and 22.2%. This study confirms that dates are a rich source of dietary fibre, and can be a valuable functional ingredient in foods that reduce the risk of chronic diseases. The study confirmed that the inexpensive non-enzymatic technique is a viable substitute for the enzymatic method for analysing dietary fibre in dry fruits.

## 1. Introduction

Dietary fibre is a diverse group of compounds resistant to digestion by digestive enzymes in the small intestine. These compounds include non-starch polysaccharides and other components such as lignin, cellulose, starch, dextrin, inulin, pectin, beta-glucan, and oligosaccharides, which play a crucial role in maintaining human health. Results from prospective cohort studies on dietary fibre in the previous decades demonstrated the vital role this macronutrient plays in reducing the risk of cardiovascular diseases [1], diabetes [2], and even gastrointestinal tract cancers [1].

Over the years, the definition of dietary fibre has evolved from non-digestible carbohydrates that are naturally present in foods to include non-digestible fibre components, either extracted or synthetic [3]. The bulk of dietary fibre in the diet comes from cereals, fruits, vegetables, legumes, and nuts; therefore, there is compositional variability in dietary fibre depending on the plant species, the part of the plant, and the plant’s maturity, as all of these factors influence the composition of dietary fibre components, such as cellulose, hemicellulose, pectin, and lignin, of the ingested food [4]. Hence, we observe variability in the health outcomes associated with dietary fibre, highlighting the importance of having reliable and cost-effective analytical methods to assess this nutrient’s composition in foods. The importance of having dietary fibre data for foods is also reinforced by the consensus in health advice across the globe that a diet rich in plant-based foods provides the best dietary protection against non-communicable diseases [4].

There are different analytical methods that can be used to determine the dietary fibre content of foods, such as proximate, gravimetric, and enzymatic-gravimetric approaches, which may or may not incorporate colorimetric or GLC/HPLC techniques. These methods allow for the determination of total dietary fibre (TDF) or TDF as separate SDF and IDF proportions, or even the individual structural elements, such as rhamnose, arabinose, xylose, mannose, uronic acid, polysaccharides, or Klason lignin. The Association of Official Agricultural Chemists (AOAC) International publishes validated methods, and some of the commonly used methods include AOAC 985.29, AOAC 991.43, AOAC 2001.03, and AOAC 2009.01. It is important to note that the method used for fibre analysis can affect the results obtained, with each method having its advantages and limitations.

AOAC 991.43 is an enzymatic-gravimetric method commonly used for measuring IDF, SDF, and TDF in foods [5]. This method involves the enzymatic hydrolysis of starch and protein, followed by the precipitation of fibrous components by aqueous ethanol. The dietary fibre residues are then weighed, and the total dietary fibre content is calculated, using with the values of residual protein and ash in the sample. ANKOM Technologies (Macedon, NY, USA) developed an automated process for the method using three heat-stable enzymes: α-amylase, protease, and amyloglucosidase [6]. Although traditional methods, such as AOAC 985.29 and AOAC 991.43, are considered gold standards, enzymes increase the cost of these analyses, thereby limiting their use in industry and research due to cost concerns [7]. However, considering all the evidence associated with the importance of including fibre in the diet, these measurements are crucial. It provides consumers with a way to assess their dietary fibre intake and make informed decisions on the nutritive value of the food they are consuming.

A key finding by Li and Cardozo suggested that enzymatic hydrolysis is not essential in foods with protein and starch contents of <2%. AOAC adopted this strategy, which was published as AOAC 993.21 [8]. Using the same rationale, a similar strategy can be employed by utilizing the automated AOAC 991.43 IDF/SDF analysis in an ANKOM dietary fibre analyser. Our previous pilot study confirmed that using AOAC 991.43 TDF analysis without enzyme hydrolysis resulted in comparable TDF measurements to those with enzymes [7]. In this study, a selection of ten date fruit varieties and other dry fruits, such as raisins, figs, and apricots, were utilized. Here we investigate whether the same strategy to exclude enzyme hydrolysis affects the accuracy of the TDF measurement as separate IDF and SDF proportions in dry fruits. The main objective of our work is to measure the TDF, IDF and SDF content in different date varieties with and without enzymatic hydrolysis using AOAC 991.43. In addition, we aim to determine the IDF and SDF proportions in the date fruit varieties and highlight their implications for human health.

## 2. Materials and Methods

### 2.1. Materials

Six date fruit varieties were obtained from local supermarkets in Al Ain, United Arab Emirates. The date fruit varieties used in the study included Lulu, Barhi, Khalas, Fard, Neghal, and Dabbas. The six varieties were chosen to encompass date fruits with varying texture profiles, ranging from soft to semi-hard to hard varieties, in order to represent date samples with significant variations in dietary fibre content [9]. Three samples of each variety, each originating from different farms, were purchased with the aim of accounting for variability in growing conditions. Chemicals such as sodium hydroxide, boric acid, hydrochloric acid, sulphuric acid, ethanol, acetone, Kjeldahl catalyst tablets, and anti-foam tablets used in the study were purchased from Sigma-Aldrich (St. Louis, MO, USA). Materials required for the ANKOM dietary fibre analyser, such as IDF and SDF filter bags, diatomaceous earth, heat-stable α-amylase, protease, and amyloglucosidase, 2-(N-morpholino)ethanesulfonic acid (MES), and Tris(hydroxymethyl) aminomethane (TRIS), were purchased from ANKOM Technologies (Macedon, NY, USA).

### 2.2. Sample Processing

The date fruit samples were deseeded, and the flesh was fine-minced using a bench-top food processor. Subsequently, the samples were desugared with 85% ethanol (ethanol: water, 85:15, *vol*/*vol*). In a laboratory shaker, 40 g fruit mince was mixed with 200 mL of the solvent. After centrifuging the mixture at 6000× *g* rpm for 10 min, the supernatant was discarded, and this process was repeated five times to eliminate the sugar. The desugared date fruit samples were dried to remove all moisture in a hot air oven at 40 °C, and the final weight of the samples was determined to account for bulk loss during the desugaring process. 

### 2.3. Dietary Fibre Analysis

The dietary fibre analysis in desugared samples was carried out using the automated dietary fibre analyser from ANKOM Technologies (Macedon, NY, USA). We opted to use the AOAC 991.43 IDF/SDF method for our study [6]. The manufacturer’s instructions were adhered to for the enzymatic method, while for the non-enzymatic method, the instrument’s enzymes were substituted with distilled water. Each sample was assessed in triplicate. In summary, 0.5 g of fruit samples were combined with MES–TRIS buffer (0.05 M, pH 8.2) for the enzymatic digestion phase. The enzymatic digestion process comprised three stages, with the first stage involving the α-amylase digestion of samples at 95 °C for 35 min. The second stage involved enzymatic digestion with protease at 60 °C for 30 min. The final phase of the process entailed digesting the samples with amyloglucosidase at 60 °C and a pH ranging between 4.0 and 4.5 for 30 min. Following enzymatic digestion, the samples were filtered via IDF filter bags, which retained the IDF components of the samples. The filtrate moved to SDF bags, where the SDF portions were precipitated with 95% ethanol. After precipitation, the mixture was again filtered by the SDF bags to retain the remaining SDF components in the sample. 

After the instrument run, the IDF and SDF bags were gathered, washed with acetone, and left to dry in a hot air oven at 105 °C overnight. The dried bags were weighed, and protein and ash contents were determined. The ash content in the IDF and SDF bags was determined by calculating the weight difference of the samples after burning them in a muffle furnace at 600 °C for 3 h. The total protein of the sample IDF and SDF portions was determined by the Kjeldahl method [10], using the general factor (6.25) to convert nitrogen to protein. The samples’ IDF (%) and SDF (%) were calculated using the following formulae.

$$\%\mathrm{IDF}=\left[\frac{\left[\left(R_{1}+R_{2}\right)/2\right]-P-A-B}{\left(M_{1}+M_{2}\right)/2}\right]\times 100$$

$$R_{1}=f_{F1}-f_{S1}$$

$$R_{2}=f_{F2}-f_{S2}$$

M1 and M2 represent the initial weight, adjusted for sugar loss (g). R1 and R2 indicate the remaining residue after analysis (g). f_F_ and f_S_ correspond to the final and initial weights, respectively, of the IDF filter bag (g). P, A, and B denote the protein value, ash content, and blank value supplied by ANKOM Technologies (Macedon, NY, USA), respectively.

$$\%\mathrm{SDF}=\left[\frac{\left[\left(R_{1}+R_{2}\right)/2\right]-P-A-B}{\left(M_{1}+M_{2}\right)/2}\right]\times 100$$

$$R_{1}=f_{F1}-f_{S1}-D_{1}$$

$$R_{2}=f_{F2}-f_{S2}-D_{2}$$

M1 and M2 denote the initial weight adjusted for sugar loss (g). R1 and R2 indicate the remaining residue after analysis (g). f_F_ and f_S_ correspond to the final and initial weights, respectively, of the SDF filter bag (g). D represents the original weight of the diatomaceous earth (g). P, A, and B denote the protein value, ash content, and blank value provided by ANKOM Technologies (Macedon, NY, USA), respectively, for both the residue and bag.

%TDF=%IDF+%SDF

### 2.4. Statistical Analyses

The statistical analysis of the experiments was conducted utilizing GraphPad Prism software version 9.1.0. Data residual were checked by the D’Agostino & Pearson test and the Shapiro-Wilk test for normality. For the comparison of enzymatic and non-enzymatic test results, Bland–Altman’s analysis and correlation plots were constructed. The IDF, SDF, and TDF contents among the different date fruit varieties were compared using ANOVA with the Tukey test. A *p*-value of ≤0.05 was considered statistically significant.

## 3. Results

### 3.1. Comparison of IDF Data between the Two Methods

**Figure 1:**
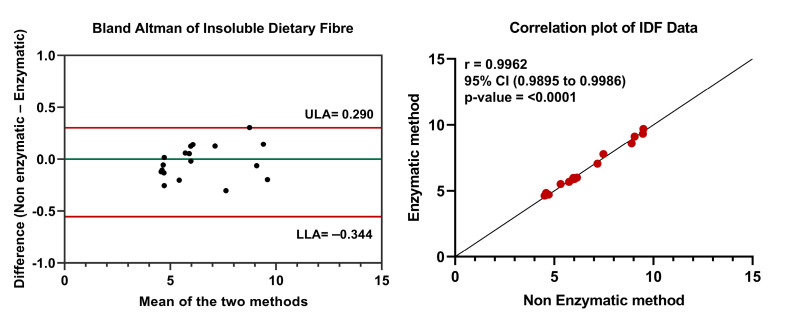
Comparison of IDF data from enzymatic and non-enzymatic methods. Bland–Altman plot was created by plotting the average and difference between the enzymatic and non-enzymatic methods. LA—Upper limit of agreement; LLA—Lower limit of agreement. Pearson’s correlation coefficient was calculated for IDF data. Statistical significance was set at *p*-value ≤ 0.05.

The IDF content in the 18 samples of date fruits measured by AOAC 991.43 with and without enzymatic digestion is provided in Table 1. Overall, the bulk of dietary fibre in date fruits was IDF. The Barhi variety recorded the lowest IDF content across the six date fruit varieties, measuring 4.51 ± 0.05% g/100 g and 4.63 ± 0.18% g/100 g with non-enzymatic and enzymatic methods, respectively. The highest IDF content was recorded in the Neghal variety, measuring 9.47 ± 0.49 and 9.69 ± 0.22 with the non-enzymatic and enzymatic methods, respectively. There was no significant difference between the results of the two methods (*p*-value 0.9644). In Figure 1, the Bland–Altman plot illustrates a high level of concurrence between the enzymatic and non-enzymatic methods for all of the samples of date fruit varieties. The disparity between the two methods is minimal on average, the limits of agreement were narrow (upper 0.290 and lower −0.344), and all measured values in the study fell within these limits. Additionally, the correlation between the two methods was examined (Figure 1). The calculated Pearson’s correlation coefficient between the enzymatic and non-enzymatic methods was 0.9962, with a 95% confidence interval between 0.9895 and 0.9986. The linear association between the two methods was significant, with a *p*-value of <0.0001.

### 3.2. Comparison of SDF Data between the Two Methods

**Figure 2:**
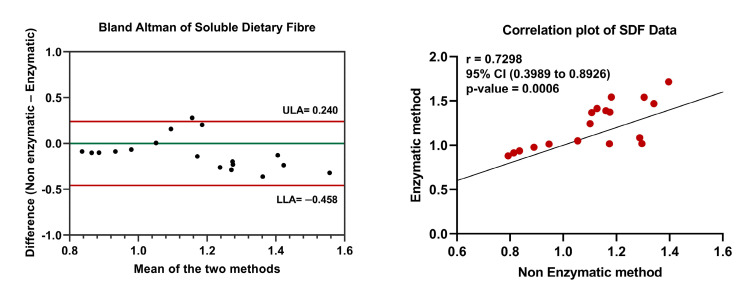
Comparison of SDF data from enzymatic and non-enzymatic methods. Bland–Altman plot was created by plotting the average and difference between the enzymatic and non-enzymatic methods. ULA—Upper limit of agreement; LLA—Lower limit of agreement. Pearson’s correlation coefficient was calculated for SDF data. Statistical significance was set at *p*-value ≤ 0.05.

The results of SDF measured in date fruits using enzymatic and non-enzymatic methods are provided in Table 1. We observed that the content of SDF in date fruits is lower than its measured IDF content. The lowest SDF content was measured in the Neghal variety, measuring 0.79 ± 0.04% g/100 g and 0.88 ± 0.05% g/100 g with the non-enzymatic and enzymatic methods, respectively. At the same time, the highest SDF content was recorded in the Barhi variety, which measured 1.30 ± 0.08% g/100 g and 1.54 ± 0.32% g/100 g with the non-enzymatic and enzymatic methods, respectively. The SDF values measured with the enzymatic method compared to the non-enzymatic method showed no significant difference (*p*-value 0.1585). The agreement between the two methods was demonstrated using a Bland–Altman plot (Figure 2), which showed a very low average difference and small limits of agreement (upper 0.240 and lower −0.458). Aside from one sample of the Lulu variety, all of the measured values were within these limits of agreement. However, the correlation between the two methods was only moderate (Figure 2). The calculated Pearson’s correlation coefficient between the enzymatic and non-enzymatic methods was 0.7298, with a larger 95% confidence interval between 0.3989 and 0.8929, compared to IDF. Nevertheless, the linear association between the two methods was significant, with a *p*-value of 0.0006.

### 3.3. Comparison of TDF Data between the Two Methods

**Figure 3:**
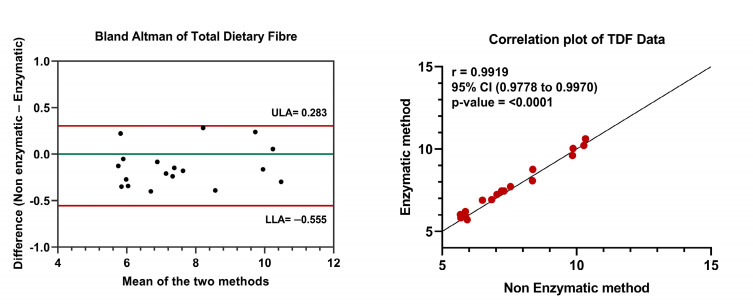
Comparison of TDF data from enzymatic and non-enzymatic methods. Bland–Altman plot was created by plotting the average and difference between the enzymatic and non-enzymatic methods. ULA—Upper limit of agreement; LLA—Lower limit of agreement. Pearson’s correlation coefficient was calculated for IDF data. Statistical significance was set at *p*-value ≤ 0.05.

The TDF content measured using both enzymatic and non-enzymatic methods showed significant variability among different date fruit varieties, as shown in Table 1. Among all the varieties, the Barhi variety recorded the lowest TDF content in the non-enzymatic method, measuring 5.67 ± 0.12% g/100 g. On the other hand, the Lulu variety was found to have the lowest TDF content in the enzymatic method, measuring 5.71 ± 0.08% g/100 g. However, the highest TDF values were observed in the Neghal variety with both non-enzymatic and enzymatic methods (10.33 ± 0.16% g/100 g and 10.63 ± 0.21% g/100 g, respectively). The *p*-value of 0.8059 indicates that there was no significant difference observed in TDF values between the enzymatic and non-enzymatic methods. The Bland–Altman plot demonstrating the agreement for TDF measurement between both methods is provided in Figure 3. From the figure, we observe that the mean discrepancy between the two methods was minimal, the range of agreement was narrow (upper limit 0.283 and lower limit −0.555), and all measured TDF values of the samples were within this range of agreement. The enzymatic and non-enzymatic methods for TDF data showed a strong correlation (Figure 3). The Pearson’s correlation coefficient between the two methods was 0.9919, with a narrow 95% confidence interval ranging from 0.9778 to 0.9970. In addition, the linear association between the two methods was also highly significant, with a *p*-value of <0.0001.

### 3.4. IDF, SDF, and TDF in Date Fruit Varieties

**Figure 4:**
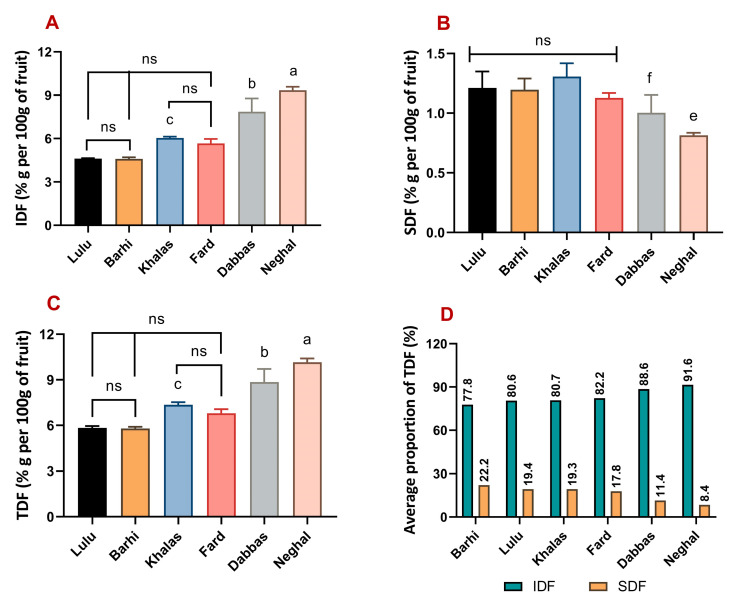
Comparison of dietary fibre content in six varieties of date fruits measured by the non-enzymatic method. (**A**) IDF data presented as mean ± s.d.; (**B**) SDF data presented as mean ± s.d.; (**C**) TDF data presented as mean ± s.d.; and (**D**) IDF and SDF data presented as an average percentage proportion of TDF. ANOVA, with multiple comparisons using the Tukey test, was used to compare the IDF, SDF, and TDF across the six date fruit varieties. Statistical significance was set at *p*-value ≤0.05. a—Neghal significant compared to all varieties; b—Dabbas significant compared to Fard, Khalas, Barhi and Lulu varieties; c—Khalas significant compared to Lulu and Barhi varieties; e—Neghal significant compared to all varieties except Dabbas; f—significant difference between Dabbas and Khalas varieties; ns—no significant difference.

The IDF, SDF, and TDF contents measured with the non-enzymatic method were used to compare their relative composition across the study’s six varieties of date fruits (Figure 4). The relative proportional average of IDF content measured in the six date fruit varieties in increasing order was: Barhi (77.8%), Lulu (80.6%), Khalas (80.7%), Fard (82.2%), Dabbas (88.6%), and Neghal (91.6%) (Figure 4D). Similarly, the average relative proportion of SDF content in increasing order was: Neghal (8.4%), Dabbas (11.4%), Fard (17.8%), Khalas (19.3%), Lulu (19.4%), and Barhi (22.1%) (Figure 4D). The Neghal variety had the highest IDF content, measuring 9.34 ± 0.25% g/100 g: significantly higher than all other varieties (Figure 4A). The second highest IDF content was measured in the Dabbas variety (7.86 ± 0.92% g/100 g), which was also significantly higher than all others except the Neghal variety. The Lulu, Fard, and Barhi varieties had comparable IDF content (4.62 ± 0.03% g/100 g, 5.66 ± 0.31% g/100 g, and 4.6 ± 0.11% g/100 g, respectively). The Khalas variety (6.05 ± 0.09% g/100 g) measured similar IDF content to that of the Fard variety; however, it was significantly higher than both the Lulu and Barhi varieties. The Barhi, Lulu, Khalas, and Fard varieties all had comparable levels of SDF content, between 1.1% g/100 g and 1.3% g/100 g (Figure 4B). These values were significantly higher than the SDF content measured in the Neghal variety (0.81 ± 0.02% g/100 g). The Dabbas variety also measured comparably low SDF content compared to the Neghal variety, with 1.0 ± 0.15% g/100 g. Consequently, the TDF content in the date fruit varieties also showed large differences, with the Neghal variety, followed by the Dabbas variety, measuring the highest (10.16 ± 0.25% g/100 g and 8.86 ± 0.86% g/100 g, respectively) (Figure 4C). The Fard and Khalas varieties had comparable dietary fibre content, and TDF content in the Khalas variety was significantly higher than that in both Lulu and Barhi varieties, which measured the lowest. 

## 4. Discussion

Date fruits are a vital agricultural crop in the middle east due to their adaptability to the harsh arid climate, and socio-cultural preferences have made date fruits one of the most popular foods in the region [11]. There are numerous date palm varieties, with around 100 grown in the United Arab Emirates alone [12]. In the Middle East, date fruits are commonly consumed year round [13]. These fruits are distinguished by a significant amount of carbohydrates (ranging from 60% to 80%), which comprise soluble sugars and dietary fibre [14]. The starch and protein contents in date fruits varies at different stages of their maturity. Starch degrades to glucose, fructose, and sucrose at the fully mature fruit stage [15]. The variations in the nutritional composition of date fruits are primarily ascribed to the dietary fibre, polyphenols, vitamins, and minerals [11]. This study investigated a new approach to analysing dietary fibre as insoluble and soluble proportions in dry fruits. Here we tested whether the enzymatic hydrolysis step was crucial in measuring IDF, SDF, and TDF in dry fruits with very low protein and starch contents using AOAC 991.43 in an ANKOM dietary fibre analyser.

When date fruits are fully ripe, their starch content is negligible, and their protein content is very low (1–1.5%). This makes them a suitable candidate for evaluating the efficacy of the non-enzymatic method in determining the content of both IDF and SDF [16]. Moreover, the dietary fibre in dates varies across the different varieties. The reported dietary fibre can range between 6.5 and 11.5% in these fruits [17]. The dietary fibre of date varieties from the UAE was reported to be between 5.5% and 9.1% [18]. More recent studies have reported TDF between 5.3 and 8.4% [19], and 5.3 and 13.4% [7]. This variability in TDF across different varieties makes date fruits a useful sample with which to measure a different range of IDF SDF, and TDF values. We used six varieties of date fruits with a range of texture profiles already reported in the literature [9]. The Neghal and Dabbas varieties have a hard texture, while the Lulu and Barhi varieties are softer. The Fard and Khalas varieties fall between the two and have a semi-hard texture. IDF, SDF, and TDF results indicate that the non-enzymatic method gave comparable measurements to the enzymatic method in all six date varieties. 

AOAC 985.29 was the first analytical method accepted as official. This enzymatic-gravimetric method was developed by Prosky et al., and it measures the TDF in dried and defatted samples with enzyme hydrolysis using three enzymes: α-amylase, protease, and amyloglucosidase [20]. AOAC 991.43 was the subsequent official method developed by Lee et al. and modified the previous method, which brought forth the possibility of measuring TDF, IDF, and SDF [21]. The measurement of TDF was similar to AOAC 985.25 and used the same three enzymes. Among the three enzymes used in both methods, α-amylase hydrolyses the α-1,4 glycosidic bonds of α-linked polysaccharides, such as starch-yielding shorter chains, e.g., dextrins. The protease enzyme hydrolyses proteins, and the amyloglucosidase enzyme hydrolyses α-1,4 and α-1,6 glycosidic bonds in starch, resulting in glucose units. 

Based on the function of each of these enzymes, we understand that they have no effect on the lignin content in the fruit samples during the enzymatic digestion step, as lignin is not a carbohydrate polymer. Lignin is a highly branched phenolic polymer made up of p-hydroxyphenyl, guaiacyl, and syringyl molecules with no regular repeating structures [22]. However, it is a significant component of the insoluble fraction of dietary fibre. Our study data indicate that the majority of dietary fibre found in date fruits is insoluble fibre. Additionally, the data support the notion that these fruits contain a significant amount of lignin. The insoluble phenolic fibres in date fruits accounted for anywhere between 1 and 5%, according to Alam et al. [23], and George et al. reported that lignin is the major component and determinant of date fruit dietary fibre [19]. Consequently, in the current study, the correlation between the enzymatic and non-enzymatic methods was very high. At the same time, the correlation between the SDF values determined by the enzymatic and non-enzymatic methods was lower than that of the IDF values. This observation may be a direct consequence of the limitation of the original AOAC 991.43, due to its inability to measure low-molecular-weight SDF. A study conducted by Tobaruela et al. compared AOAC 991.43 to the new method, AOAC 2011.25, for measuring dietary fibre content in fruits [24]. The study reported that the IDF content of fruits, quantified by both methods, showed no significant difference. One key difference between the two methods is their ability to measure low-molecular-weight SDF portions. Only AOAC 2011.25 measured the low-molecular-weight SDF, which can be attributed to various factors, such as the type and purity of enzymes, incubation time and temperature, and precipitation conditions. These factors may have affected the SDF quantification, resulting in final values with significant differences. Detailed analysis of date fruit fibres has reported that dates contain SDF sources, such as fructan, pectin, galactomannan, arabinoxylan, and β-glucan in different degrees of variability between the different varieties [19]. Low-molecular-weight SDF components may have been underestimated in the enzymatic and non-enzymatic methods in the study. In the Bland–Altman analysis of SDF data, one sample of the Lulu variety was above the limit of agreement, which could be a direct consequence of the AOAC 991.43’s limitation to account for low molecular weight SDF fractions. It is important to note that the Lulu variety was one of the varieties that reported a higher content of SDF. In general, despite the variations observed, they did not impact the consistency between the two methods or the linear association in the TDF correlation between the enzymatic and non-enzymatic methods. Consequently, the current study’s findings support the conclusions from our prior research, which reported a strong consistency between the enzymatic and non-enzymatic methods for TDF measurement in date fruits and other dried fruits, such as apricots, figs, and raisins [7].

The concept of dietary fibre is not new since its relevance to health, and its extraction from animal feed and forages, was recorded in Germany as early as the 1850s [25]. Various definitions of dietary fibre have been suggested and debated for decades. Recently, a consensus has been forming around the definition, adopted by CODEX in 2009 [26], which broadly describes the types of fibre from naturally occurring food, those obtained from raw food material via extraction, and synthetic carbohydrate polymers. Among these three broad categories, there is significant diversity at the chemical composition and physical structure levels. Classification of dietary fibres based on chemical composition is beneficial from an analytical standpoint, since it increases the analysis’s robustness, accuracy, and repeatability [22]. Other than structural classification, the solubility of the dietary fibre within the gastrointestinal tract during digestion is commonly used to describe dietary fibre types. This classification is helpful because solubility influences its functionality concerning health outcomes [3].

Since the reliability of the non-enzymatic method was established, the dietary fibre content in date fruit varieties analysed by this method was used for comparison. The TDF in date fruits varied between 5.83 ± 0.13% g/100 g and 10.16 ± 0.25% g/100 g, which is comparable to the range reported by Al-Shahib and Marshall (6.4–11.5%) [17], Habib et al. (5.52–9.11%) [18], and Ali et al. (5.4% to 13.6%) [7]. In this study, the calculated proportions of IDF and SDF were 91.6–77.8% and 8.4–22.2%. An earlier investigation of SDF and IDF proportions in Tunisian date varieties reported comparable data: 84–94% insoluble and 6–16% soluble [27]. Cellulose, hemicelluloses, and lignin are the chief components of the IDF proportion in date fruits, while pectin, fructooligosaccharides, inulin, galactomannan, and β-glucan constitute the SDF proportion [19]. It is reported that the pectin content varies anywhere between 0.5% and 3.9% in date fruits [17]. Moreover, the composition of dietary fibre in date fruits changes with the ripening process. The percentages of pectin, hemicellulose, cellulose, and lignin decreases significantly as the fruit reaches the fully ripened stage [17]. George et al. stated that lignin was the major component of dietary fibre in fully ripe date fruits, comprising a significant proportion of TDF [19]. 

Various nutritional functionalities are associated with date fruits’ IDF and SDF proportions. SDF sources, such as pectin and fructans that are present in date fruits, can increase the viscosity of the food due to its water-holding capacity and consequently decrease the rate of gastric emptying and nutrient absorption, which can help satiety [28] and lower cholesterol levels [29], enhance glucose tolerance [30], and increase insulin sensitivity [31]. Additionally, pectin is also known to improve the serum lipid profile [29,32] and stimulate bile acid secretion [32]. However, the hydration capacity of dietary fibre does not depend on solubility; in fact, IDF sources with large molecular weight can hold water and increase stool volume in order to speed up the rate of faecal passage. Since the significant proportion of dietary fibre in date fruits consist of IDF fractions, their health implications are primarily derived from their interaction with colonic microbiota. IDF sources present in date fruits, such as cellulose, hemicellulose, and lignin, are fermented by gut microbiota, a process which consequently generates short-chain fatty acids, which have numerous associated health benefits. Within the intestinal lumen, these compounds promote the growth of beneficial gut microbes, increase colonic sodium and water absorption, inhibit tumour formation [33], and stimulate mucosal cell proliferation [34]. On a systemic level, SFCA generated from IDF content can inhibit cholesterol synthesis [3] and even positively modulate systemic inflammation [35].

## 5. Conclusions

Our study results indicate that AOAC 991.43 can be used to accurately measure the contents of IDF, SDF, and TDF in dry fruits with low protein and starch contents without the need for enzymatic digestion. This finding makes the non-enzymatic method of AOAC 991.43 an economical alternative for analysing dietary fibre in dry fruits. Moreover, our results indicate that date fruits have high dietary fibre content, especially IDF. The measured contents of IDF, SDF, and TDF varied significantly across the six varieties in the study and, based on their compositional characteristics, has a wide range of favourable implications for human health. One of the main limitations of this study was that we did not include other dry fruits, such as apricots, figs, or raisins, into which these findings could be translated. This limitation can be addressed in future collaborative studies that assess the reliability of the non-enzymatic method between different laboratories.

## Figures and Tables

**Table 1:** Analysis of dietary fibre in different varieties of date fruits using enzymatic gravimetric method with and without enzymatic digestion.

	Insoluble Dietary Fibre ^a^		Soluble Dietary Fibre ^a^		Total Dietary Fibre ^a^	
	Non-Enzymatic	Enzymatic	Non-Enzymatic	Enzymatic	Non-Enzymatic	Enzymatic
Lulu 1	4.64 ± 0.13	4.69 ± 0.13	1.30 ± 0.06	1.02 ± 0.07	5.93 ± 0.08	5.71 ± 0.08
Lulu 2	4.58 ± 0.31	4.84 ± 0.17	1.29 ± 0.07	1.08 ± 0.05	5.87 ± 0.37	5.92 ± 0.13
Lulu 3	4.64 ± 0.33	4.77 ± 0.14	1.05 ± 0.01	1.05 ± 0.05	5.69 ± 0.33	5.82 ± 0.09
Fard 1	5.32 ± 0.02	5.52 ± 0.18	1.18 ± 0.11	1.37 ± 0.20	6.50 ± 0.10	6.90 ± 0.23
Fard 2	5.74 ± 0.08	5.69 ± 0.06	1.10 ± 0.15	1.24 ± 0.13	6.84 ± 0.17	6.93 ± 0.13
Fard 3	5.93 ± 0.06	5.87 ± 0.10	1.11 ± 0.15	1.37 ± 0.33	7.04 ± 0.10	7.24 ± 0.24
Dabbas 1	7.48 ± 0.18	7.78 ± 0.02	0.89 ± 0.16	0.98 ± 0.09	8.37 ± 0.11	8.76 ± 0.10
Dabbas 2	7.18 ± 0.09	7.06 ± 0.43	1.17 ± 0.17	1.02 ± 0.03	8.36 ± 0.09	8.07 ± 0.41
Dabbas 3	8.90 ± 0.01	8.60 ± 0.01	0.95 ± 0.11	1.01 ± 0.04	9.85 ± 0.10	9.61 ± 0.03
Khalas 1	6.03 ± 0.15	5.91 ± 0.13	1.18 ± 0.13	1.54 ± 0.18	7.21 ± 0.26	7.45 ± 0.28
Khalas 2	5.96 ± 0.18	5.98 ± 0.20	1.34 ± 0.35	1.47 ± 0.20	7.30 ± 0.19	7.45 ± 0.40
Khalas 3	6.14 ± 0.07	6.00 ± 0.04	1.40 ± 0.16	1.72 ± 0.12	7.54 ± 0.13	7.72 ± 0.13
Barhi 1	4.72 ± 0.23	4.70 ± 0.13	1.13 ± 0.11	1.41 ± 0.14	5.85 ± 0.25	6.12 ± 0.11
Barhi 2	4.51 ± 0.05	4.63 ± 0.18	1.16 ± 0.13	1.39 ± 0.10	5.67 ± 0.12	6.02 ± 0.19
Barhi 3	4.56 ± 0.10	4.67 ± 0.18	1.30 ± 0.08	1.54 ± 0.32	5.86 ± 0.17	6.21 ± 0.49
Neghal 1	9.50 ± 0.14	9.69 ± 0.22	0.84 ± 0.03	0.94 ± 0.07	10.33 ± 0.16	10.63 ± 0.21
Neghal 2	9.06 ± 0.24	9.12 ± 0.27	0.81 ± 0.00	0.91 ± 0.08	9.87 ± 0.24	10.03 ± 0.33
Neghal 3	9.47 ± 0.49	9.33 ± 0.09	0.79 ± 0.04	0.88 ± 0.05	10.27 ± 0.45	10.21 ± 0.10

The contents of IDF, SDF, and TDF are expressed as %g/100 g of fruit, and the data are presented as mean ± standard deviation. Each sample was analysed in triplicate, and the data sets were compared using the t-test, with statistical significance set at a *p*-value of ≤0.05. ^a^ No significant difference between the two methods.

## Data Availability

The data that support the findings of this research are available from the corresponding author upon reasonable request.
